# LncRNA Lncbate1 promotes lipid synthesis in the white adipose tissue during aging by targeting the miR-455-5p―ACSS1 axis

**DOI:** 10.1038/s41514-026-00337-1

**Published:** 2026-06-19

**Authors:** Jing Chen, Ning Yang, Yifan Zhang, Qianqian Yao, Chengfu Yuan

**Affiliations:** 1 College of Basic Medical Science, Hubei University of Chinese Medicine, Wuhan, China; 2 Hubei Key Laboratory of Tumor Microenvironment and Immunotherapy, China Three Gorges University, Yichang, China; 3 College of Basic Medical Science, China Three Gorges University, Yichang, China; 4 Third-grade Pharmacological Laboratory on Traditional Chinese Medicine, State Administration of Traditional Chinese Medicine, China Three Gorges University, Yichang, China

**Keywords:** Biochemistry, Cardiology, Cell biology, Diseases, Endocrinology, Physiology

## Abstract

Lipid metabolism is closely related to aging, and its disorders can lead to obesity, insulin resistance, cardiovascular diseases, and various other conditions. The accumulation of lipids caused by increased lipid synthesis is key in the development of obesity, but the molecular mechanisms of increased lipid synthesis remain to be thoroughly studied, especially in the context of aging. In this work, we used in vivo and in vitro models to demonstrate that the long noncoding RNA Lncbate1 contributes to obesity by promoting lipid accumulation. Specifically, Lncbate1 increases lipid synthesis by downregulating the expression of miR-455-5p to increase the expression of the lipid synthesis gene *ACSS1*. Our findings highlighted Lncbate1 as a likely target for the intervention of aging-related obesity.

## Introduction

Aging is now recognized as a multifactorial biological process driven by geroscience-defined hallmarks such as genomic instability, telomere attrition, epigenetic alterations and mitochondrial dysfunction, among others^1^. These interconnected alterations progressively disrupt tissue homeostasis and promote age-related metabolic disturbances. In particular, lipid metabolism disorders in the aging process lead to blood lipid disorders, chronic low-grade inflammation of the adipose tissue, and obesity ^2,3^. Correcting the lipid metabolism abnormalities in the aging process to reduce lipid deposition is expected to mitigate the obesity caused by aging.

The acyl-CoA synthetase short chain family members 1 (ACSS1) and 2 (ACSS2) play a key role in lipid synthesis in multiple pathologies^4^. For example, exogenously stressed cancer cells can utilize exogenous acetate to produce acetyl-CoA through ACSS1/2, to enable the de novo synthesis of fat and upregulate key genes for lipid metabolism^5^. Studies have also shown that ACSS2-deficient mice under high-fat diet challenge display lower body weight gain and attenuated hepatic steatosis^6^. Human hepatocellular carcinoma with high ACSS1/2 expression exhibits increased expression of fatty acid synthase (*FASN*), a key gene for lipid synthesis^7^. However, within the context of aging, the impact of ACSS1/2 on lipid anabolism remains to be further examined.

Noncoding RNA (ncRNA) accounts for about 98% of primate genomic transcripts. Long noncoding RNAs (lncRNAs), defined as ncRNAs consisting of more than 200 nucleotides^8,9^, are closely associated with metabolic diseases^10–12^ and the regulation of adipogenesis^13,14^. The lncRNA Lncbate1 has a powerful impact on the establishment and maintenance of the identity and thermogenic capacity of the brown adipose tissue (BAT), and some studies have shown that Lncbate1 can bind to the heterogeneous nuclear ribonucleoprotein U and regulate the production of BAT^15,16^. However, the impact of Lncbate1 on the white adipose tissue is unclear, and it remains uncertain if Lncbate1 is involved in lipid metabolism disorders during aging.

Previous studies have reported that multiple lncRNAs are dysregulated in liver tissue during aging^17,18^. In this work, we demonstrated that Lncbate1 was significantly upregulated in the epididymal white adipose tissue (eWAT) in aging mice. Through rigorous experimentation, we demonstrated that Lncbate1 worked as a competitive endogenous RNA (ceRNA)^19^ to promote lipid synthesis in senescent adipocytes via the miR-455-5p/ACSS1 axis, thus showing that it is an important target for managing the metabolism of the adipose tissue during aging.

## Results

### Lipid synthesis and senescence

**Fig. 1:**
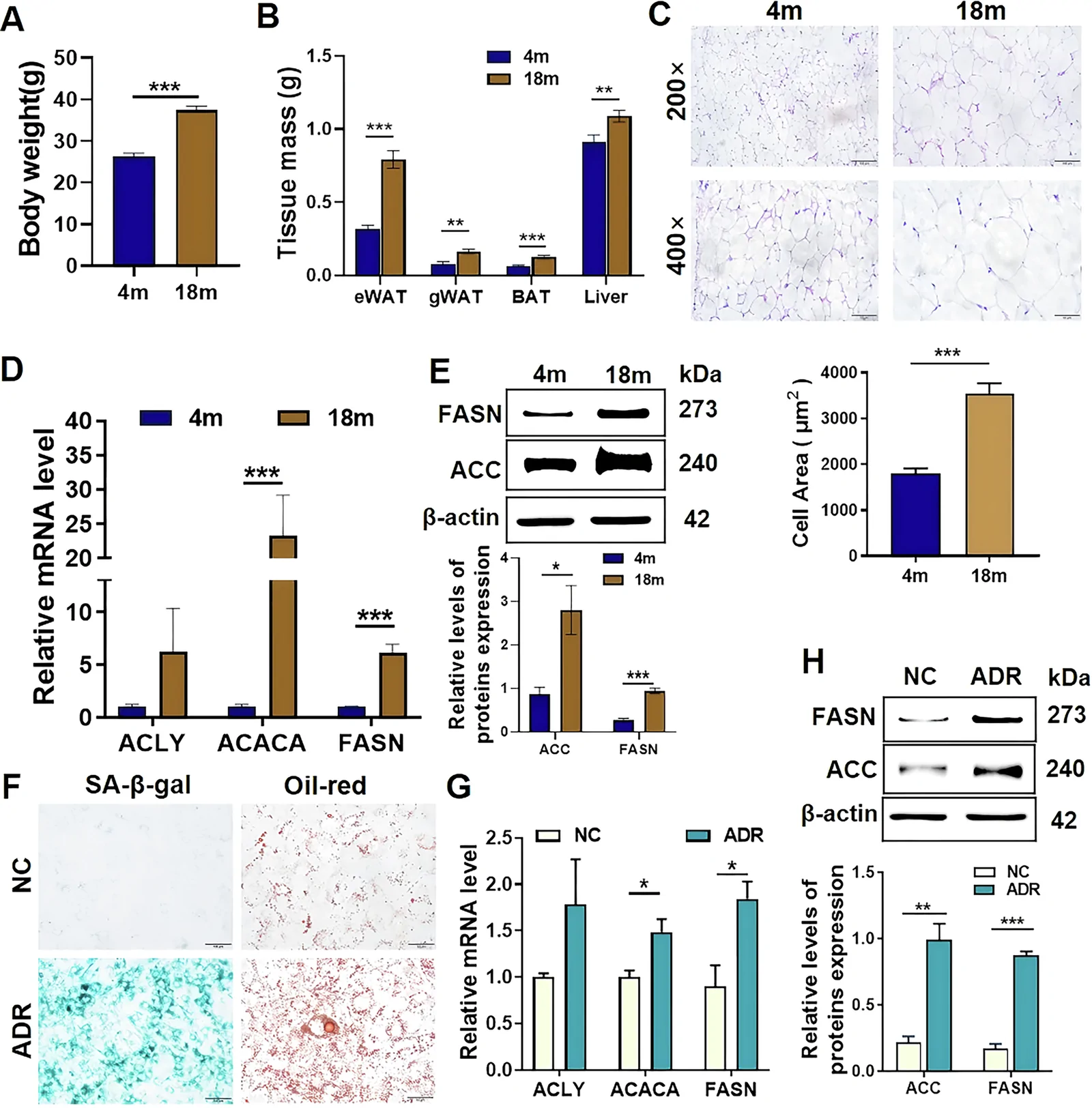
Lipid synthesis is increased due to aging and senescence. **A** Body weight of adult (4-month-old) and aging (18-month-old) mice (*n* = 8). **B** Tissue weight (eWAT, gWAT, BAT, and liver) of adult and aging mice (*n* = 8). **C** H&E staining of the eWAT (scale bar = 50 μm). **D** The mRNA expression of *ACLY*, *ACACA*, and *FASN* in the eWAT (*n* = 3). **E** The protein levels of ACC and FASN in the eWAT (*n* = 5). (**F**) β-gal staining and oil red O staining of mature adipocytes (NC) and senescent adipocytes (ADR) derived from 3T3-L1 cells. Scale bar: 100 μm for β-gal, 50 μm for oil red O. **G** The mRNA expression of *ACLY*, *ACACA*, and *FASN* in mature adipocytes (NC) and senescent adipocytes (ADR) derived from 3T3-L1 cells (*n* = 3). **H** The protein levels of ACC and FASN in mature adipocytes (NC) and senescent adipocytes (ADR) derived from 3T3-L1 cells (*n* = 3). Data are presented as the mean ± SEM. * *P* < 0.05, ** *P* < 0.01, *** *P* < 0.001.

Aging clearly increased lipid accumulation in the mouse model. Compared to the adult mice (4-month-old), the aging mice (18-month-old) had significantly higher body weight, liver tissue weight, and adipose tissue weight (eWAT, gWAT, and BAT) (Fig. 1A, B). The H&E staining of eWAT revealed significant morphological changes in the adipocytes of aging mice, featured by an increased adipocyte diameter and a reduced cell count per unit area (Fig. 1C). The expression of acetyl-CoA carboxylase (*ACC*) and *FASN*, both pivotal genes in lipid biosynthesis, were markedly elevated in the eWAT of aging mice (Fig. 1D), and the protein levels of ACC and FASN exhibited a consistent trend (Fig. 1E).

Cellular senescence is one of the hallmarks of aging and contributes to the functional decline of WAT^20^. To model aging-related changes in adipocytes in vitro, we established an ADR-induced senescence model. In this model, ADR induction caused a noticeable increase in cell size and a flattened morphology in β-gal staining compared to the negative control (NC) (Fig. 1F). Oil red O staining further showed that senescent adipocytes exhibited greater lipid droplet accumulation and aggregation than NC (Fig. 1F). Regarding the gene expression profile, the senescent adipocytes displayed an upregulation in the mRNA levels of *ACACA* and *FASN*, although the mRNA level of *ACLY* did not exhibit a similar upregulation (Fig. 1G). The protein levels of ACC and FASN were also elevated in the senescent cells (Fig. 1H).

### Lipid synthesis in senescent adipocytes with Lncbate1 knockdown

**Fig. 2:**
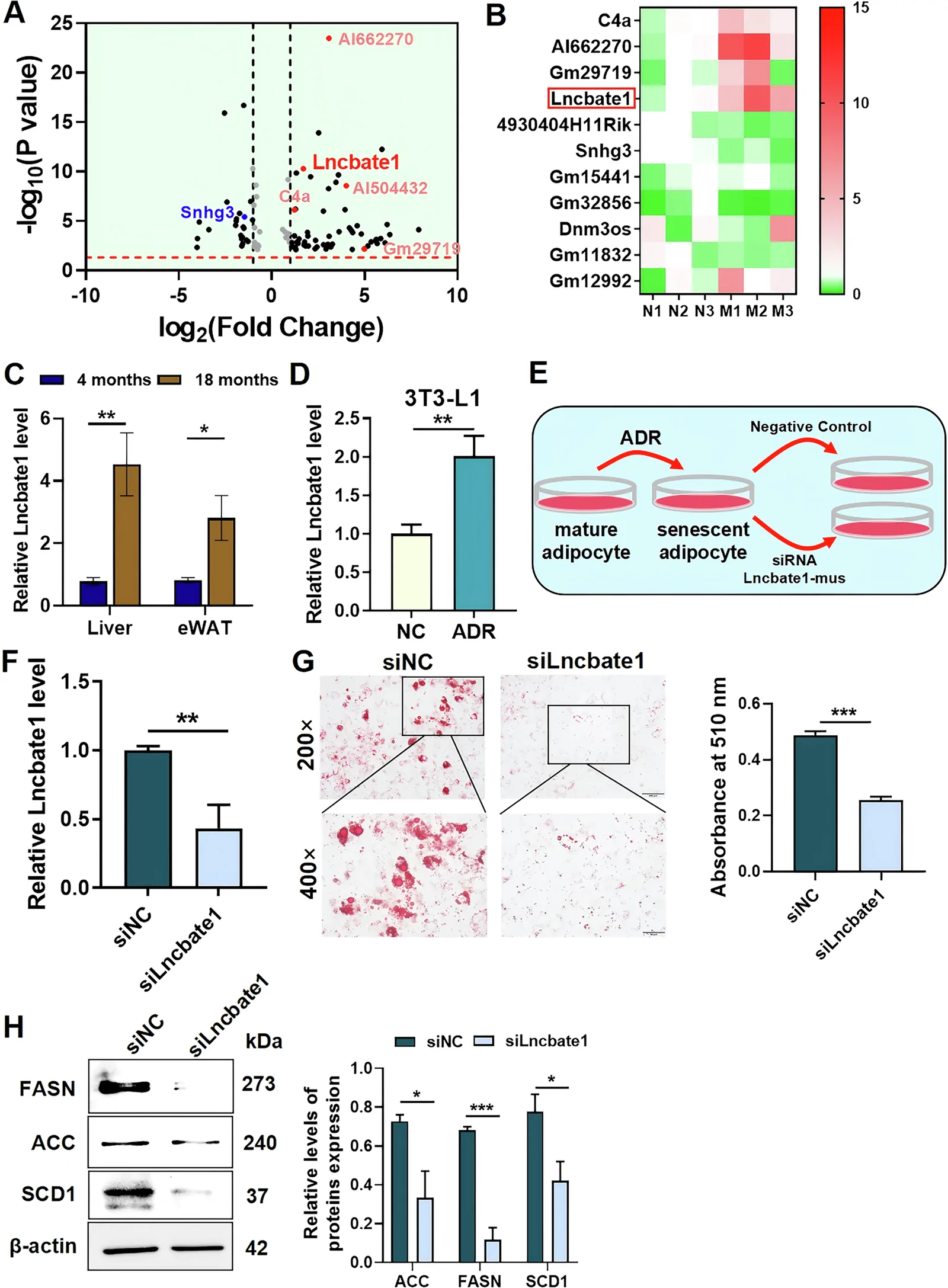
The increased lipid synthesis in senescent adipocytes is associated with Lncbate1. **A** Identification of differentially expressed LncRNAs in eWAT between aging and adult mice from the Volcano map of the RNA-seq data. **B** Heat map analysis of differentially expressed LncRNAs. The expression level of Lncbate1 **C** in the eWAT and liver of the animal models (*n* = 8) and **D** in 3T3-L1 cells (*n* = 7). **E** Cell models of Lncbate1 knockdown (siLncbate1) and negative control (siNC). **F** The expression of Lncbate1 in the cell models (*n* = 3). **G** Representative oil red O staining images and quantification of the cell models (*n* = 3). The scale bar is 100 μm for 200× images and 50 μm for 400× images. **H** The protein levels of SCD1, ACC, and FASN in the cell models (*n* = 3). Data are presented as the mean ± SEM. * *P* < 0.05, ** *P* < 0.01, *** *P* < 0.001.

Eleven noncoding RNAs were differentially expressed between the aging mice (18-month-old) and the adult mice (4-month-old) according to RNA-seq of eWAT (Fig. 2A). The expression of Lncbate1 was enhanced in the aging mice (Fig. 2B). The RT-qPCR experiment also confirmed that the expression of Lncbate1 was significantly upregulated in the liver tissue and the eWAT of the aging mice (Fig. 2C) and in the senescent adipocytes (Fig. 2D).

Cell experiments were then carried out to transfect senescent adipocytes with siLncbate1 (Fig. 2E), to thus examine the impact of Lncbate1 knockdown on lipid synthesis in senescent adipocytes. The transfection significantly downregulated the expression of Lncbate1 (Fig. 2F), proving that siLncbate1 successfully silenced Lncbate1 in senescent adipocytes. The oil red O staining revealed that the knockdown of Lncbate1 significantly reduced the number and size of lipid droplets in the senescent adipocytes. Additionally, the absorbance of the eluate at 510 nm, which correlates with lipid content, was significantly decreased (Fig. 2G). The protein levels of ACC, FASN and SCD1 were also significantly reduced by the Lncbate1 knockdown, which indicated decreased lipid synthesis in the senescent adipocytes after the Lncbate1 knockdown (Fig. 2H).

### Interaction between Lncbate1 and miR-455-5p

**Fig. 3:**
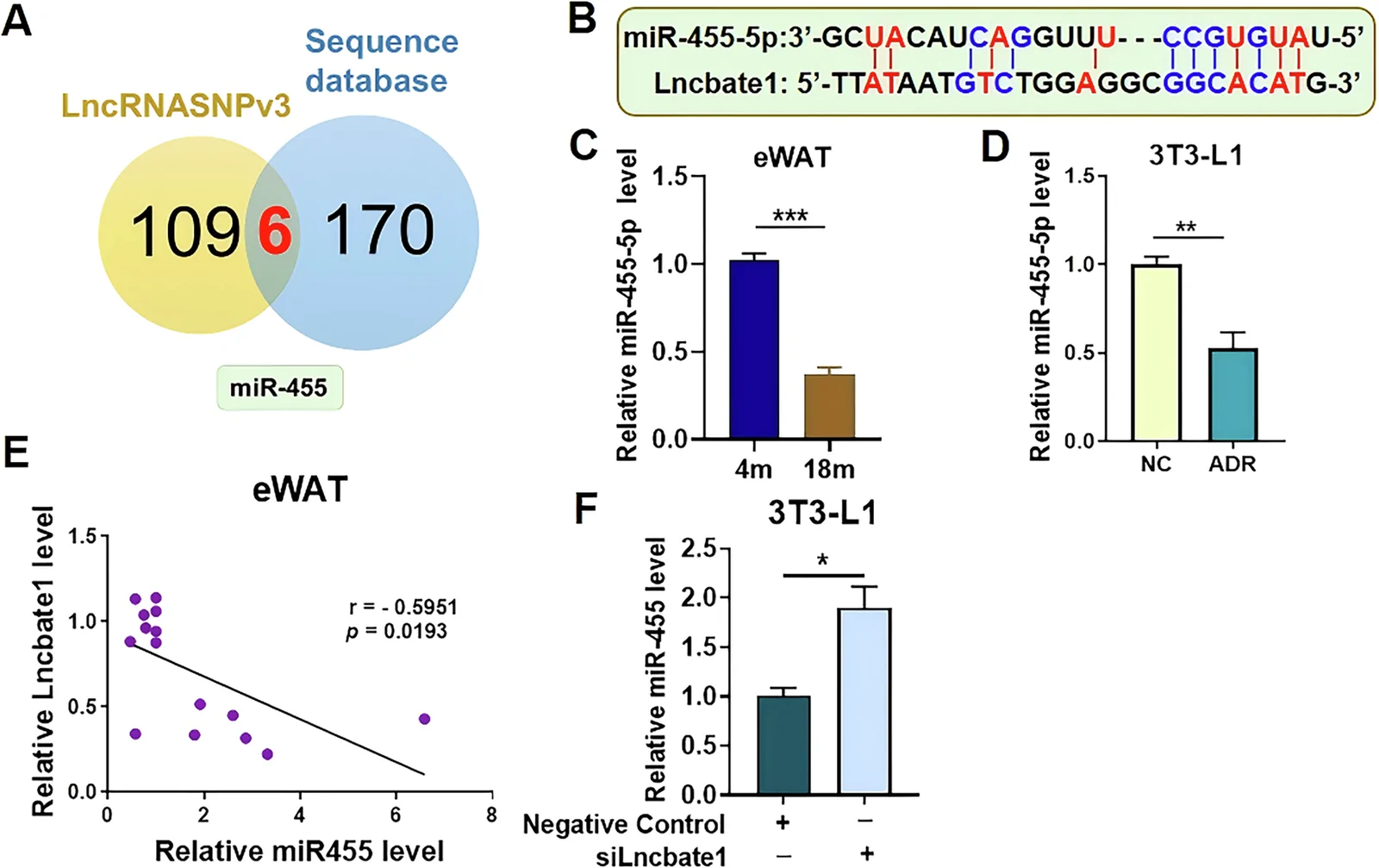
Lncbate1 is involved in lipid synthesis by negatively regulating miR-455-5p. **A** Potential downstream miRNA targets of Lncbate1. **B** Binding sites between Lncbate1 and miR-455-5p. The mRNA expression of miR-455-5p in **C** the eWAT (*n* = 8) and **D** 3T3-L1 cells (*n* = 7). **E** Pearson correlation analysis of Lncbate1 and miR-455-5p in the eWAT. **F** The mRNA expression of miR-455-5p in senescent adipocytes after Lncbate1 knockdown (*n* = 3). Data are presented as the mean ± SEM. * *P* < 0.05, ** *P* < 0.01, *** *P* < 0.001.

The subcellular localization of lncRNA determines its biomolecular mechanisms. The iLoc-lncRNA prediction suggests that Lncbate1 is distributed in the cytoplasm (Fig. S1). We therefore hypothesized that Lncbate1 might regulate gene expression through a competitive ceRNA mechanism, as cytoplasmic lncRNAs are known to modulate mRNA levels by binding and sequestering microRNAs (miRNAs)^21^. To identify candidate miRNAs that could mediate the effects of Lncbate1 on lipid metabolism, we analyzed differential miRNA expression in the eWAT of adult mice and aging mice by RNA-seq. The results showed that many miRNAs were expressed differentially. We also used the bioinformatics website LncRNASNPv3 to predict possible miRNAs downstream of Lncbate1. Six miRNAs, including miR-455-5p, were both within the sequencing results and the LncRNASNPv3 prediction (Fig. 3A). There were 12 putative complementary binding sites between miR-455-5p and Lncbate1 (Fig. 3B). The RT-qPCR results showed that the expression of miR-455-5p was significantly reduced in the senescent adipocytes both in vivo (Fig. 3C) and in vitro (Fig. 3D). In the animal studies, an inverse relationship existed between the levels of Lncbate1 and miR-455-5p (Fig. 3E). In addition, the senescent adipocytes with Lncbate1 knockdown exhibited significantly enhanced miR-455-5p expression (Fig. 3F). It is likely that miR-455-5p was negatively regulated by Lncbate1.

### miR-455-5p regulates lipid accumulation

**Fig. 4:**
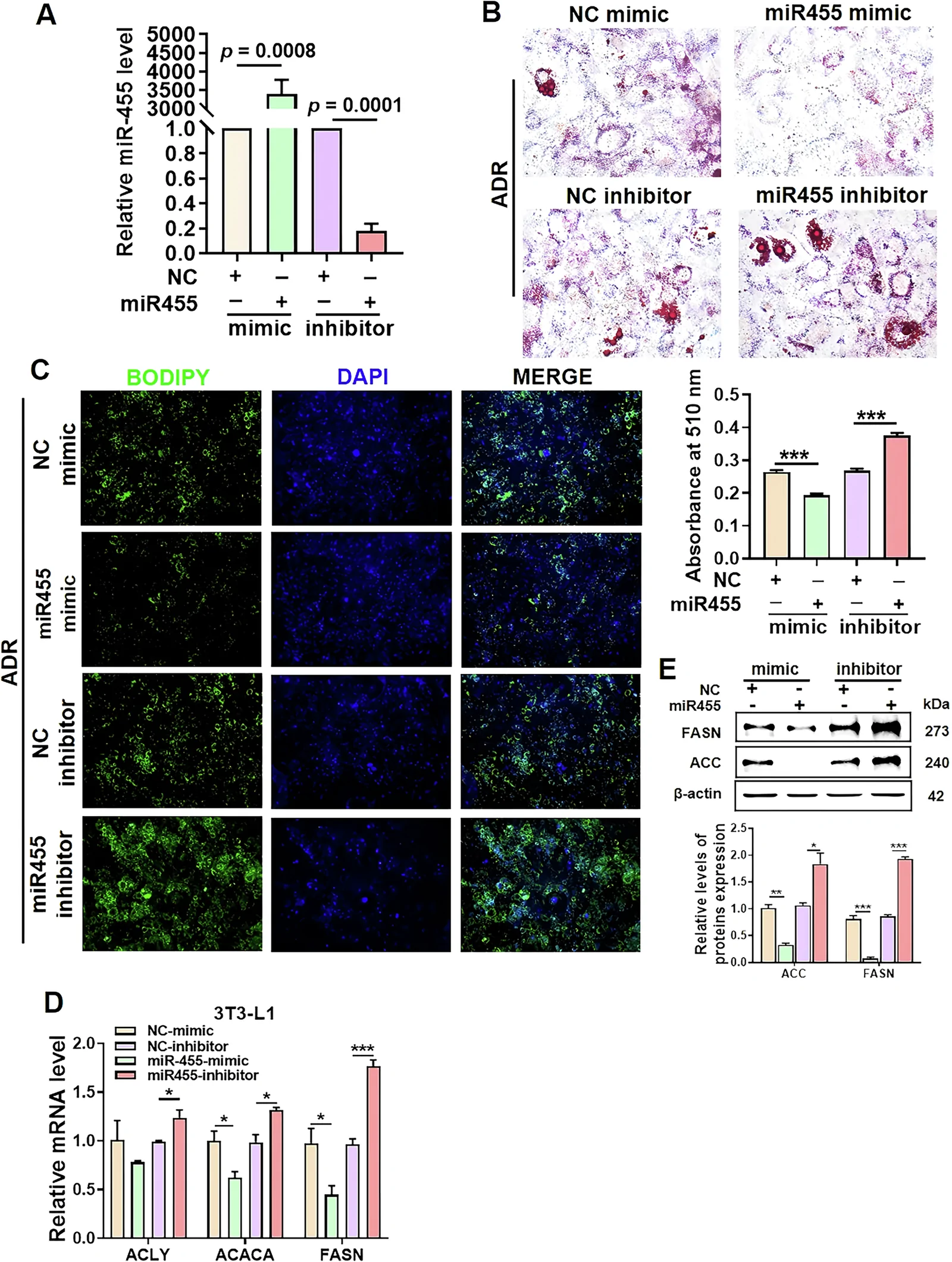
miR-455-5p reduces lipid synthesis in senescent adipocytes. **A** The miR-455-5p expression in senescent adipocytes after transfection with miR-455-5p mimic or inhibitor (*n* = 3). **B** Representative oil red O staining images and quantification and **C** BODIPY staining of senescent adipocytes after miR-455-5p editing (*n* = 3). Scale bar: 50 μm for oil red O, 200 μm for BODIPY. **D** The mRNA expression of *ACLY*, *ACACA*, and *FASN* in senescent adipocytes after miR-455-5p editing (*n* = 3). **E** The protein levels of ACC and FASN in senescent adipocytes after miR-455-5p editing (*n* = 3). Data are presented as the mean ± SEM. * *P* < 0.05, ** *P* < 0.01, *** *P* < 0.001.

To investigate the involvement of miR-455-5p in lipid synthesis, 3T3-L1 cells were transfected with miR-455-5p mimic and inhibitor (Fig. 4A). The cells transfected with the miR-455-5p mimic had a notable reduction in lipid droplets, but the miR-455-5p inhibitor increased the accumulation of lipid droplets, giving a greater count and larger sizes of the lipid droplets in oil red O staining (Fig. 4B). The BODIPY staining results corroborated these findings (Fig. 4C), hence suggesting a strong link between miR-455-5p and lipid metabolism. The mRNA expression and protein levels of ACC1 and FASN both exhibited a consistent trend (Fig. 4D, E).

### miR-455-5p targets ACSS1

**Fig. 5:**
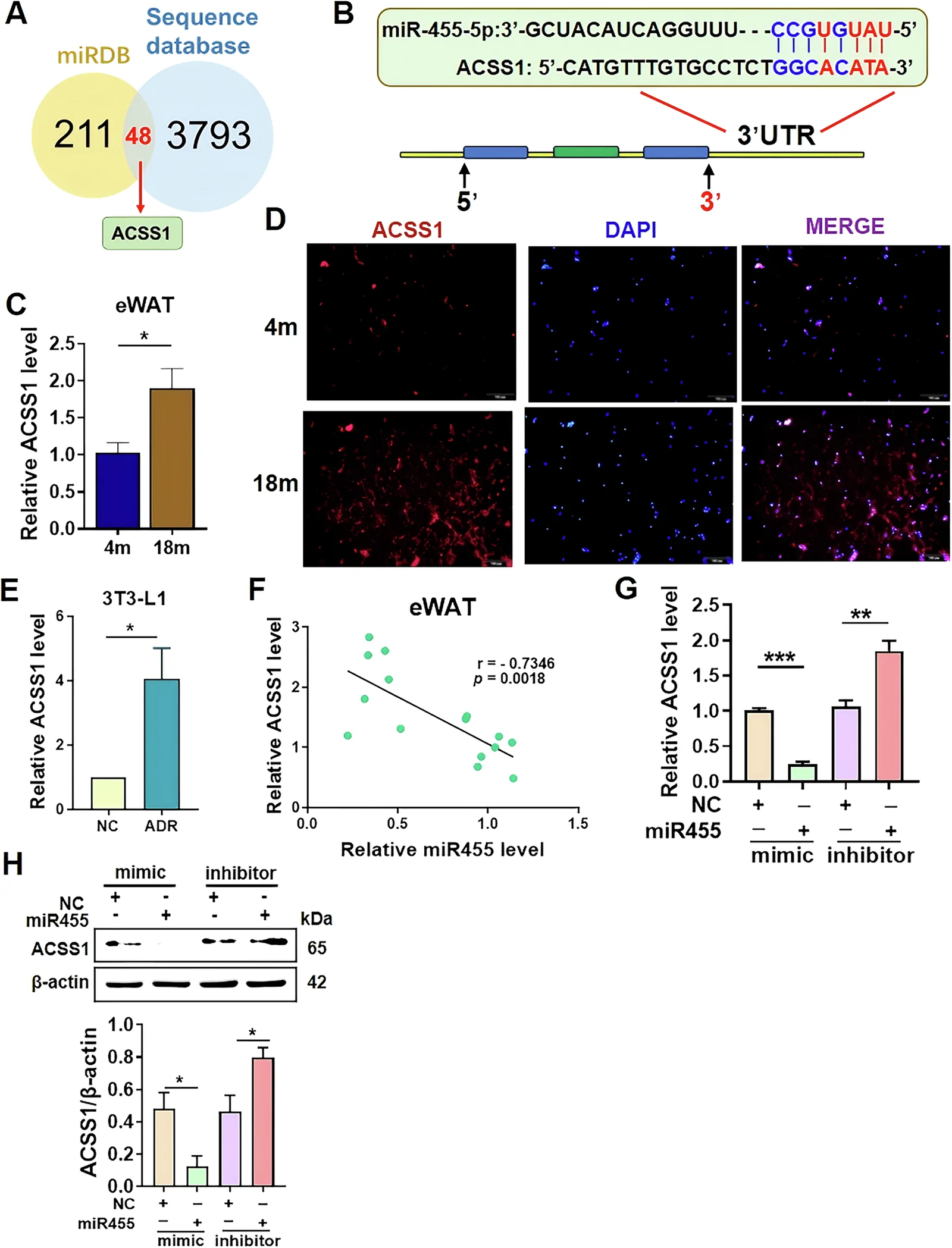
miR-455-5p inhibits lipid synthesis by targeting ACSS1. **A** Potential downstream mRNA targets of miR-455-5p. **B** Binding sites between miR-455-5p and *ACSS1*. **C** The mRNA expression of *ACSS1* in the eWAT (*n* = 8). **D** The immunofluorescence detection of ACSS1 in the eWAT (scale bar = 100 μm). **E** The mRNA expression of *ACSS1* in senescent (ADR) and mature (NC) adipocytes (*n* = 3). **F** Pearson correlation analysis of miR-455-5p and *ACSS1* expression in the eWAT. **G** The mRNA expression of *ACSS1* in senescent adipocytes after miR-455-5p editing (*n* = 3). **H** The protein levels of ACSS1 in senescent adipocytes after miR-455-5p editing (*n* = 3). Data are presented as the mean ± SEM. * *P* < 0.05, ** *P* < 0.01, *** *P* < 0.001.

Of all the downstream genes of miR-455-5p predicted by miRDB, 48 were detected in our sequencing results, including *ACSS1* (Fig. 5A). Bioinformatic analysis predicted seven putative binding sites between *ACSS1* and miR-455-5p (Fig. 5B). In the animal models, the aging mice had significantly higher levels of *ACSS1* expression (Fig. 5C) and denser red fluorescence areas of ACSS1 in the eWAT (Fig. 5D). The data obtained from immunofluorescence assays agreed with the immunohistochemistry findings (Fig. S2). Furthermore, an inverse relationship was observed between the expression of *ACSS1* and miR-455-5p (Fig. 5F).

In the cellular models, the senescent adipocytes exhibited a significant upsurge in *ACSS1* expression (Fig. 5E). The mRNA expression and protein levels of ACSS1 decreased after the transfection of the miR-455-5p mimic but increased after the transfection of the miR-455-5p inhibitor (Fig. 5G, H). These data support *ACSS1* as a likely downstream target of miR-455-5p in adipocytes.

### Lncbate1 promotes lipid synthesis via the miR-455-5p―ACSS1 axis

**Fig. 6:**
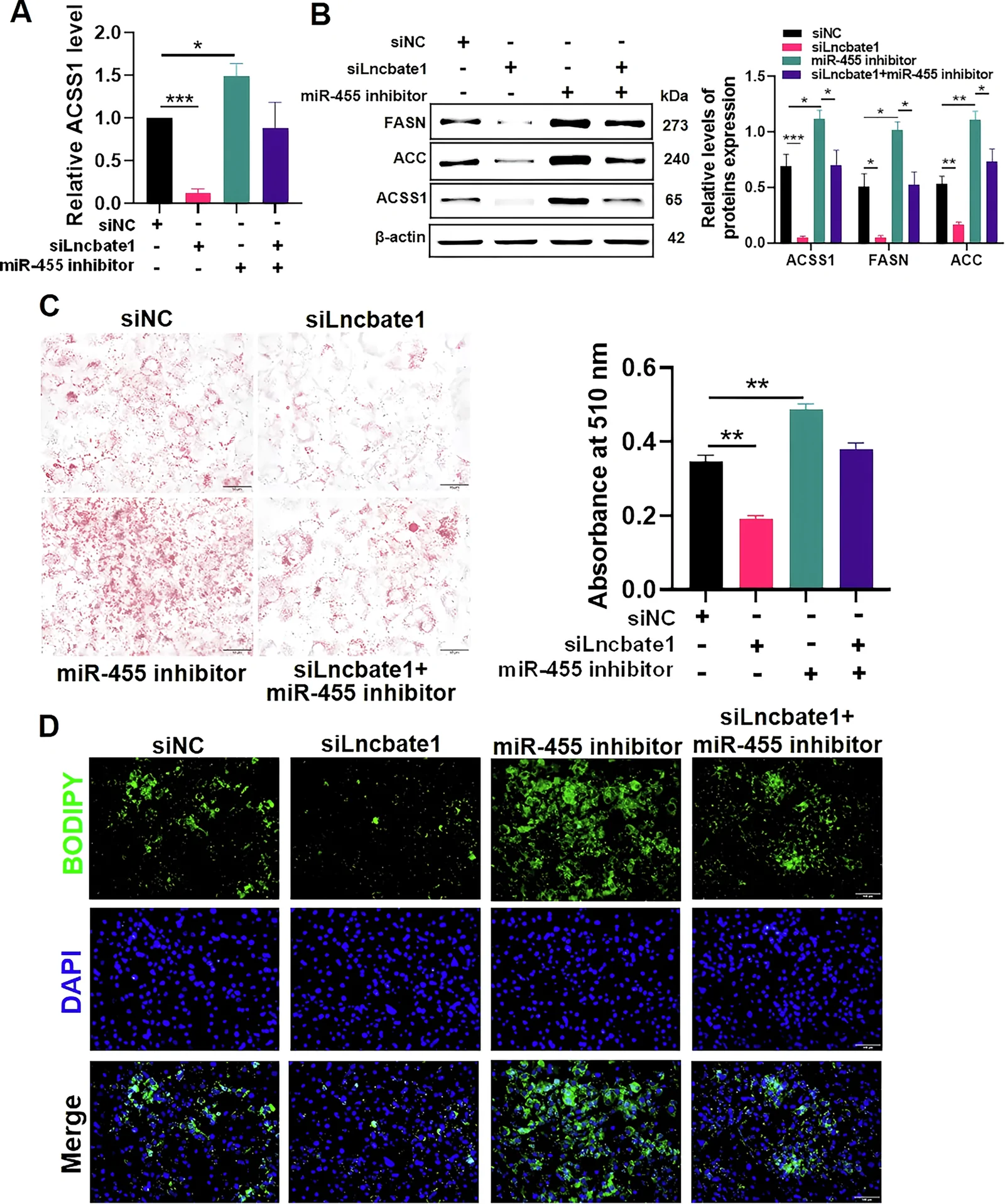
Lncbate1 promotes lipid synthesis in senescent adipocytes via the miR-455-5p/ACSS1 pathway. **A** The mRNA expression and **B** the protein levels of ACSS1 in senescent adipocytes cells after co-transfection of miR-455-5p inhibitor and siLncbate1 (*n* = 3). **C** Representative oil red O staining images and quantification and **D** BODIPY staining of senescent adipocytes after co-transfection of miR-455-5p inhibitor and siLncbate1 (*n* = 3). Scale bar: 50 μm for oil red O, 100 μm for BODIPY. Data are presented as the mean ± SEM. * *P* < 0.05, ** *P* < 0.01, *** *P* < 0.001.

It appeared plausible from the above that Lncbate1 interacted with the miR-455-5p/ACSS1 axis to promote lipid synthesis. To verify this hypothesis, we transfected both the miR-455-5p inhibitor and the siLncbate1 to senescent adipocytes and examined the lipid synthesis metrics. The elevation of *ACSS1* expression caused by the miR-455-5p inhibitor was clearly offset by the Lncbate1 knockdown (Fig. 6A, B), and the lipid droplet aggregation visualized by oil red O staining and BODIPY staining also suggested that siLncbate1 and miR-455-5p inhibitor counteracted each other (Fig. 6C, D). Hence, miR-455-5p appears to be an important mediator of Lncbate1-dependent lipid synthesis, at least in part, through the miR-455-5p/ACSS1 axis.

## Discussion

Aging tends to accentuate obesity, and the changes in lipid metabolism and metabolic disorders are essential in aging-related diseases^22^. Due to the prevalence of obesity in the older population, aging-related metabolic diseases are increasingly becoming a public health problem^23,24^. Thorough explorations of the underlying mechanisms of obesity in the aging process are essential to preventing and treating aging-related metabolic diseases.

The role of lncRNAs in regulating lipid metabolism disorder has been well documented^25,26^, and many studies have shown how lncRNAs regulate various biological processes through transcription and post-transcription^27^. The WAT contains abundant lncRNAs. The dynamic crosstalk between lncRNA and mRNA in the WAT plays an important role in regulating the function of the aging adipose tissue^28,29^, and lncRNAs can also promote the de novo synthesis of fatty acids^30^.

Lncbate1 is a noncoding RNA located on mouse chromosome 8 and is highly enriched in the mouse BAT and eWAT, but its relationship with lipid anabolism during aging remains unreported to date. In this work, using RNA-seq, we found a significant upregulation of Lncbate1 in the eWAT of aging mice. The RT-qPCR results confirmed that Lncbate1 was highly expressed in the eWAT of aging mice. The in vitro experiments proved that in senescent fat cells, Lncbate1 increased the number of lipid droplets and the aggregation of lipid droplets. The key enzymes in lipogenesis, including FASN, SCD1, and ACC1, were also affected by Lncbate1. The high Lncbate1 levels correlated to the characteristics of lipid synthesis and were predictors of lipid deposition in senescent adipocytes.

Since lncRNAs commonly exert their regulatory function by sponging miRNAs^29,31,32^ and Lncbate1 was localized in the cytoplasm, we then screened the downstream miRNA targets of Lncbate1 by bioinformatics analysis, and miR-455-5p was identified as a candidate. Further functional experiments verified that Lncbate1 could sponge miR-455-5p to promote lipid synthesis in senescent adipocytes. We then combined the results of bioinformatics analysis and RNA-seq to study the target gene of the Lncbate1/miR-455-5p pathway in senescent adipocytes, and we found that *ACSS1* was a downstream effector of the Lncbate1/miR-455-5p axis. *ACSS1* has been identified as a gene that promotes fat synthesis, and the acetyl-CoA synthetase (ACS) family enzymes are known to participate in hepatic lipid metabolism by synthesizing acetate^6^.

Our data identified *ACSS1* as a direct downstream target of the Lncbate1/miR-455-5p axis, yet we also observed significant changes in the expression of *FASN* and *ACC1*. As we did not identify canonical miR-455-5p binding sites within the *FASN* or *ACC1* transcripts, their regulation is likely indirect. ACSS1 catalyzes the conversion of acetate to acetyl-CoA, a fundamental substrate for lipogenesis^33^. It is well established that intracellular acetyl-CoA levels can influence the activity and expression of key lipogenic transcription factors, such as *SREBP-1* and *ChREBP*^34^. Therefore, we postulate that the Lncbate1-mediated upregulation of *ACSS1* increases the acetyl-CoA pool, which subsequently drives the expression of *FASN* and *ACC1* through these metabolic sensing pathways, rather than through direct miRNA targeting.

Finally, the context-dependent function of Lncbate1 is worth noting. Previous studies have characterized Lncbate1 as a regulator of BAT, showing that it supports thermogenesis and suppresses white fat genes^15^. However, in our RNA-seq analysis of eWAT from aged mice, classical brown fat markers (e.g., Ucp1, Pgc1a) were expressed at negligible levels and were not induced, which indicates a loss of thermogenic potential in this tissue. Thus, the functional role of Lncbate1 seems to be highly plastic and dependent on the cellular environment. In the context of aged white adipose tissue, Lncbate1 appears to function predominantly as a cytoplasmic ceRNA driving lipid storage via the miR-455-5p/*ACSS1* axis, distinct from its nuclear transcriptional role in maintaining brown adipocyte identity in younger animals.

This study has several limitations that warrant discussion. First, while our results demonstrate a functional link between Lncbate1, miR-455-5p, and ACSS1, we did not perform direct binding assays, such as luciferase reporter assays or RNA immunoprecipitation (RIP), to confirm the physical interaction between these molecules. Similarly, we did not utilize site-directed mutagenesis to disrupt predicted binding sites or rescue experiments with Lncbate1 overexpression to definitively isolate the ceRNA mechanism. However, the convergence of bioinformatic predictions, inverse expression patterns in vivo and in vitro, and the reciprocal phenotypic effects observed in our knockdown and inhibitor experiments provide strong evidence for this regulatory axis.

**Fig. 7:**
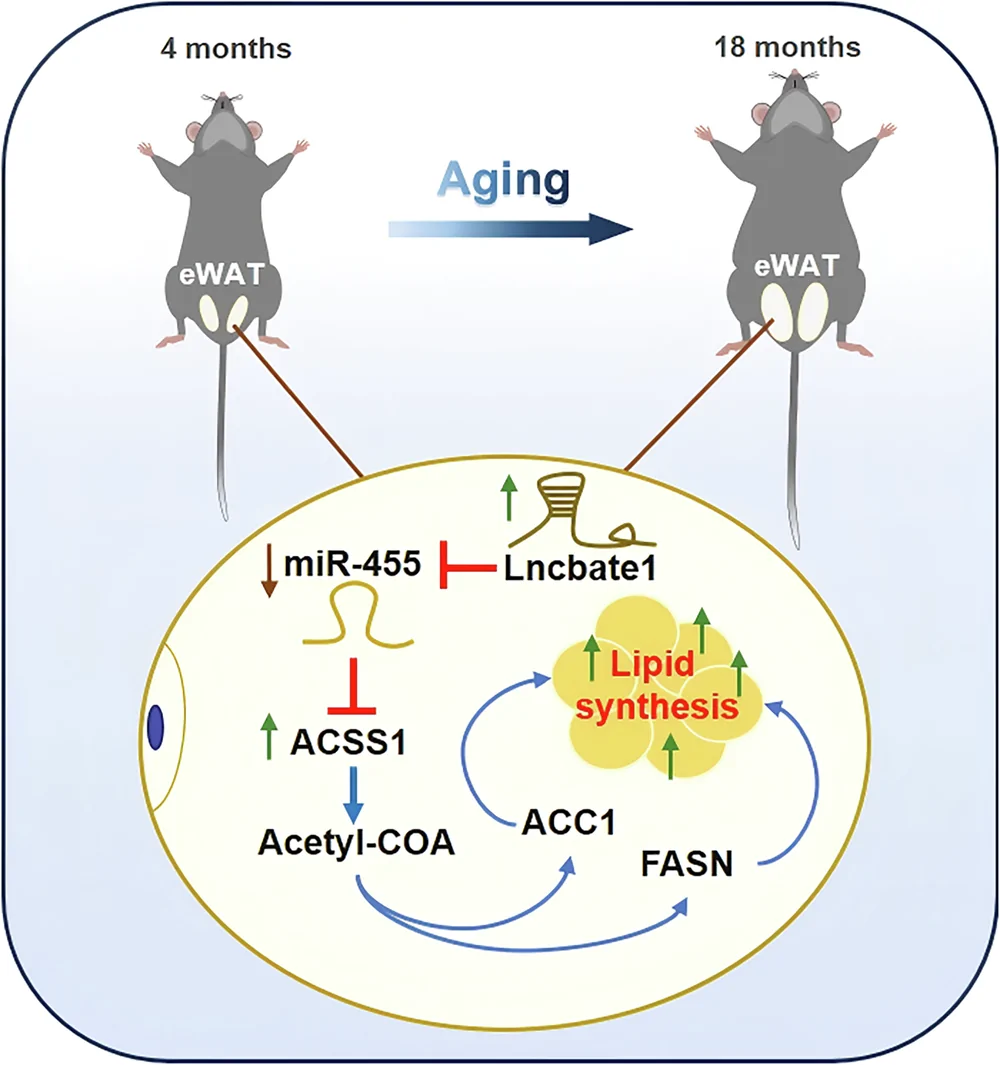
Lncbate1 promotes age-associated adipose lipogenesis via the miR-455-5p/ACSS1 pathway.

Second, our in vitro experiments relied on ADR-induced senescence in 3T3-L1 adipocytes. While senescence is a key component of aging, this chemical model may not fully recapitulate the complex physiological alterations of natural adipose tissue aging. Furthermore, we identified senescent cells primarily through SA-β-gal staining and morphological assessment; although these are standard markers, the inclusion of additional molecular markers such as p16INK4a or p21CIP1 would have strengthened the characterization. Finally, RNA-seq identified multiple miRNAs differentially expressed in aging eWAT. We prioritized miR-455-5p based on its established relevance to lipid metabolism, but other candidates such as miR-34a-5p or miR-449a-5p may also contribute to the phenotype and merit future investigation. Overall, our results provide preliminary evidence suggesting that Lncbate1 likely promotes adipogenesis via the miR-455-5p/ACSS1 pathway (Fig. 7).

## Methods

### Animals

Six-week-old male C57/BL6J mice were randomly divided into two groups each having eight mice. They were raised in an SPF level animal room at 21 ± 1 °C with a 12 h/12 h light and dark cycle. The mice in the adult group were raised to 4 months old and those in the aging group to 18 months old. They were fed a standard chow diet containing 2.8% fat, 61.5% carbohydrate, and 25.7% protein throughout the study; the diet was obtained from the Experimental Animal Center of China Three Gorges University (Yichang, China). No mice died during the experiment. At the end of the experiment, all mice were first anesthetized with an intraperitoneal injection of 20% urethane and 10 mg/kg xylazine and then euthanized by an overdose of the same anesthetic. The BAT, eWAT, and liver tissue were retrieved, flash frozen in liquid nitrogen, and stored at −80 °C until use. All animal experiments were approved by the Animal Protection and Utilization Management Committee of China Three Gorges University (2019080D, March 4, 2019) and strictly followed the National Institutes of Health Guide for the Care and Use of Laboratory Animals.

### Cells culture and differentiation

3T3-L1 cells were sourced from ATCC. Three media were used to culture the cells. The first was high-glucose Dulbecco’s Modified Eagle Medium (DMEM; Gibco, USA) with 10% fetal bovine serum (FBS) and 1% penicillin/streptomycin. The second was the first medium supplemented with 1 μM dexamethasone (DEX), 0.5 mM 3-isobutyl-1-methylxanthine (IBMX), 5 μg/mL insulin, 125 μM indomethacin, and 1 mM rosiglitazone. The third was the first medium supplemented only with 5 μg/mL insulin.

The cells were initially incubated at 37 °C under a humid atmosphere with 5% CO_2_ in the first medium, which was refreshed daily, until confluence was reached. They were then incubated in the second medium for 48 h to trigger differentiation, and finally incubated in the third medium, which was refreshed every 2 days, until the formation of abundant lipid droplets. Mature adipocytes were then collected and treated with 0.2 μM adriamycin (ADR) for 48 h to induce in vitro senescence^35^.

### Cells transfection

3T3-L1 cells were cultivated at 37 °C under a humid atmosphere containing 5% CO_2_ in DMEM supplemented with 10% FBS. They underwent transfection after progressing through the stages of maturation, differentiation, and senescence induction. The transfection process used a miR-455-5p inhibitor, a miR-455-5p mimic, and siLncbate1, all sourced from Genepharma (Shanghai, China). The transfection agent was Lipofectamine 8000, obtained from Beyotime (Shanghai, China). The experiments were carried out strictly in accordance with the guidelines provided by the manufacturer.

### RNA oligo sequence

The sequence of siLncbate1 was GGUACAUGCCUCUGGUCUUTT (5′ to 3′) and its antisense sequence was AAGACCAGAGGCAUGUACCTT (5′ to 3′). The sequence of miR-455-5p mimic was UAUGUGCCUUUGGACUACAUCG and its antisense sequence was AUGUAGUCCAAAGGCACAUAUU (5′ to 3′). The sequence of miR-455-5p inhibitor was CGAUGUAGUCCAAAGGCACAUA (5′ to 3′).

### β-Galactosidase (β-gal) staining

After the cells were washed once with PBS for 3 min, they were immobilized in the β-galactosidase (β-gal) fixative solution (1 mL) at ambient temperature for 15 min, and the β-gal fixative was removed by washing with PBS three times. The cells were then incubated at 37 °C overnight, washed with PBS once, and examined under a standard light microscope (OLYMPUS CKX53; Tokyo, Japan).

### Oil red O staining

After the cells were washed with PBS twice, they were fixed in a 4% solution of paraformaldehyde for 20–30 min, then washed with PBS twice before incubation in a freshly prepared oil red O solution for 10 min. To remove any excess stain, the cells were briefly immersed in 60% isopropanol for 1 min. Finally, the cells were washed with PBS 2 to 5 times until the liquid was clear and colorless. The lipid droplets dyed red were observed using a light microscope (OLYMPUS CKX53).

### BODIPY staining

Cells were washed with PBS twice, fixed with 4% paraformaldehyde for 15 min, then washed with PBS twice and incubated for 20 min in a freshly prepared BODIPY staining solution at 1:8000 dilution. The stained cells were washed with PBS twice before incubation in DAPI solution for 5 min, then washed with PBS three times and sealed with an anti-fluorescence quencher. The fluorescence was observed under a light microscope (OLYMPUS CKX53).

### Hematoxylin & eosin (H&E) staining

The adipose tissue stripped from the epididymis of the euthanized mice was fixed in 4% paraformaldehyde for 24 h, dehydrated using an alcohol gradient, embedded in paraffin, and finally sliced into 4 μm sections. The sections were stained with H&E according to standard protocols, then observed using a normal light microscope (OLYMPUS CKX53). Cell areas were analyzed using ImageJ.

### Quantitative real-time PCR (RT-qPCR)

The eWAT or the cells were lysed thoroughly with TRIzol in an EP tube. After the addition of chloroform (200 μL) to the lysate, the tube was shaken vigorously for 30 s, kept on ice for 5 min, and centrifuged at 4 °C and 12,000 × *g* for 15 min. The supernatant was aspirated into a new EP tube, shaken with an equal volume of isopropanol, and kept on ice for 10 min. The mixture was then centrifuged at 4 °C and 12,000 × *g* for 15 min, and the supernatant was discarded. The pellets were washed by the addition of 75% ethanol (1 mL), and the mixture was centrifuged at 4 °C and 7500 × *g* for 5 min. Finally, the supernatant was discarded, and the residue was dried and dissolved in 10–20 μL DEPC water. The concentration and purity of the RNA were then measured on a NanoDrop 2000 spectrometer (Thermo Fisher; Waltham, MA, USA).

The RNA (1 μg) was reverse transcribed into cDNA by PCR amplification using Vazyme’s HiScript III kit (Nanjing, China). The RT-PCR reaction solution (10 μL) consisted of SYBR, the forward primer, the reverse primer, cDNA, and RNase-free H_2_O. The PCR amplification was carried out in the dark and included 40 cycles of predenaturation at 95 °C for 10 min, denaturation at 95 °C for 15 s, and annealing at 60 °C for 1 min. Table 1 lists the primers used in this experiment. The relative expression levels were quantified using the 2^−ΔΔCT^ method. The internal control was β-actin when mRNA and LncRNA were analyzed and U6 when miRNA was analyzed.

**Table 1:** The primer sequences used for real-time PCR

Name	Primer	Sequence
β-actin	Forward	5′-TGCTGTCCCTGTATGCCTCT-3′
	Reverse	5′-TTTGATGTCACGCACTATTT-3′
ACLY	Forward	5′-GAAGCTGACCTTGCTGAACC-3′
	Reverse	5′-GAAGCTGACCTTGCTGAACC-3′
ACACA	Forward	5′-GCCTCTTCCTGACAAACGAG-3′
	Reverse	5′-TGACTGCCGAAACATCTCTG-3′
FASN	Forward	5′-TGGGTTCTAGCCAGCAGAGT-3′
	Reverse	5′-ACCACCAGAGACCGTTATGC-3′
Lncbate1	Forward	5′-ACACGTGCCACTGCCTAC-3′
	Reverse	5′-AAGGTGATGCTGAGCGGG-3′
ACSS1	Forward	5′-TCTTCCCTGCGCACACTG-3′
	Reverse	5′-GGGAGGATCTCTGCCCCA-3′
mmu-miR-455-5p	Forward	5′-GTCGTATCCAGTGCAGGGTCCGAGGTATTCGCACTGGATACGACCGATGT-3′
	Reverse	5′-GGGTGTATGTGCCTTTGGACT-3′
U6	Forward	5′-CTCGCTTCGGCAGCACA-3′
	Reverse	5′-AACGCTTCACGAATTTGCGT-3′
Lnc-AI662270	Forward	5′-GGTTTTCGGCGCCATGTG-3′
	Reverse	5′-GACAGTCGGGGTTCTGCC-3′
Lnc-Gm29719	Forward	5′-TCCGAGTGCCGTGTGTTC-3′
	Reverse	5′-TGCTGCACTGGCTCTCAC-3′
Lnc-Gm15441	Forward	5′-TCAGGGACTTGCGCATCG-3′
	Reverse	5′-CAGTCGAGTGCCTGGTGG-3′
Lnc-C4a	Forward	5′- ACGGTTTGCGCTCATGG -3′
	Reverse	5′- CAAGGGAACGGAGCTGGG -3′
Lnc- Dnm3os	Forward	5′-CCTCCTTCCTGCTTGCCC-3′
	Reverse	5′-AACAAGGGGGAGAGGGCA-3′
Lnc-Gm11832	Forward	5′-GCACGATGCTGAGAGGCT-3′
	Reverse	5′-CTGCTGCGAGCTGGTTCT-3′
Lnc-Snhg3	Forward	5′-GCGTCGCTCTCTTGGTGT-3′
	Reverse	5′-GGCAGAAAAGCTTGCCCC-3′
Lnc-Gm32856	Forward	5′-GCGCGCTATCTCGTGACT-3′
	Reverse	5′-TTCTGCTGCAGTGCCTCC-3′
Lnc-Gm12992	Forward	5′-TTCCTGCCAAGAGATGCTGG-3′
	Reverse	5′-CAACACTGGGCTTTGCAGAC-3′
Lnc- 4930404H11Rik	Forward	5′-CCATGAGGTGCAGGGGTG-3′
	Reverse	5′-TTCTTGTGCCCCTCTGCC-3′

### RNA sequencing

Sequencing was carried out based on reported protocols^36^. Differentially expressed lncRNAs between adult mice and aging mice were identified by RNA-seq (BGI Genomics Co., Ltd; Shenzhen, China) and analyzed using the DESeq R package. To control the false discovery rate (FDR), *P* values were adjusted using the Benjamini–Hochberg method. The lncRNAs were considered to express differentially if |log_2_[fold change (FC)]| > 1 and FDR < 0.05.

### Western blot

Total proteins were extracted from the tissues or cells using a protein extraction kit (Solarbio; Beijing, China), and the concentration of the proteins was determined using the BCA protein assay. The proteins (30 μg) were separated by SDS-PAGE, and the bands were transferred onto PVDF membranes (Millipore; Burlington, MA, USA). The membranes were blocked with 5% non-fat milk at room temperature for 1 h and incubated at 4 °C overnight with the primary antibodies listed in Table 2. Afterwards, the membranes were washed with Tris-buffered saline containing 0.1% Tween-20 (TBST) and incubated for 2 h at room temperature with the corresponding secondary antibodies (Table 2). The protein expression was visualized using an enhanced chemiluminescence substrate (Millipore; Burlington, MA, USA) with β-actin as the internal standard. The blots were analyzed quantitatively using ImageJ.

**Table 2:** The antibodies used for Western blot analysis

Antibody	Catalog number and supplier
anti-rabbit ACSS1	A15007, ABclonal
anti-rabbit ACC	A15606, ABclonal
anti-rabbit FASN	A19050, ABclonal
anti-rabbit β-actin	AC026, ABclonal
anti-rabbit SCD1	A16429, ABclonal
anti-rabbit IgG, HRP-linked antibody	7074, Cell Signaling Technology

### Bioinformatics

The LncRNASNPv3 database (https://www.gonglab.hzau.edu.cn/lncRNASNP3/) was used to predict the microRNAs possessing the binding sites for Lncbate1. The results were amalgamated and visualized using Venn diagrams, and microRNAs of high confidence were then chosen. The miRDB database (http://www.mirdb.org/) was used to predict the potential target genes of miR-455-5p.

### Statistics

Data was analyzed using SPSS 22.0 (IBM Corp.; Armonk, NY, USA) and visualized using GraphPad Prism 8 (GraphPad Software; Boston, MA, USA). The experimental results were expressed as mean ± SEM. For two-group comparisons, if the data followed a normal distribution and had homogeneous variances, an unpaired two-tailed Student’s *t*-test was used; otherwise, the Mann–Whitney *U* test was applied. For multiple group comparisons, if the data met parametric assumptions, one-way analysis of variance (ANOVA) followed by Tukey’s post hoc test was used. If the data did not meet such assumptions, Kruskal–Wallis test followed by Dunn’s post hoc test was applied. Differences were considered statistically significant when *P* < 0.05.

## Supplementary information

Supplementary Information

## Data Availability

Additional data are available on request.
