# Supplementary material for: LncRNA Lncbate1 promotes lipid synthesis in the white adipose tissue during aging by targeting the miR-455-5p―ACSS1 axis
Source: NPJ Aging. 2026 Jun 19;12(1):156. doi: 10.1038/s41514-026-00337-1 (PMC13755445; doi:10.1038/s41514-026-00337-1)
Supplement: Supplementary file 1 [file 41514_2026_337_MOESM1_ESM.pdf]

Supplementary Figure S1

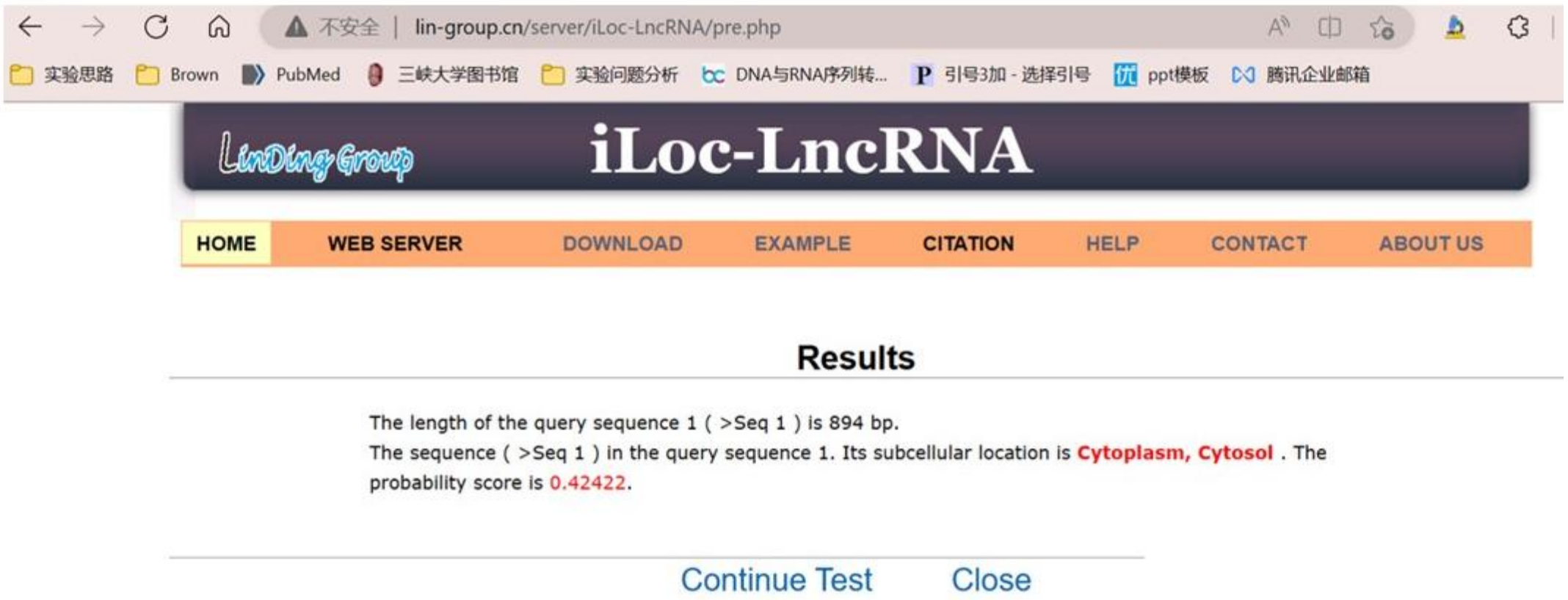

Fig. S1. Prediction of the subcellular localization of Lncbate1 (http://lin-group.cn/server/iLoc-LncRNA/).

Supplementary Figure S2

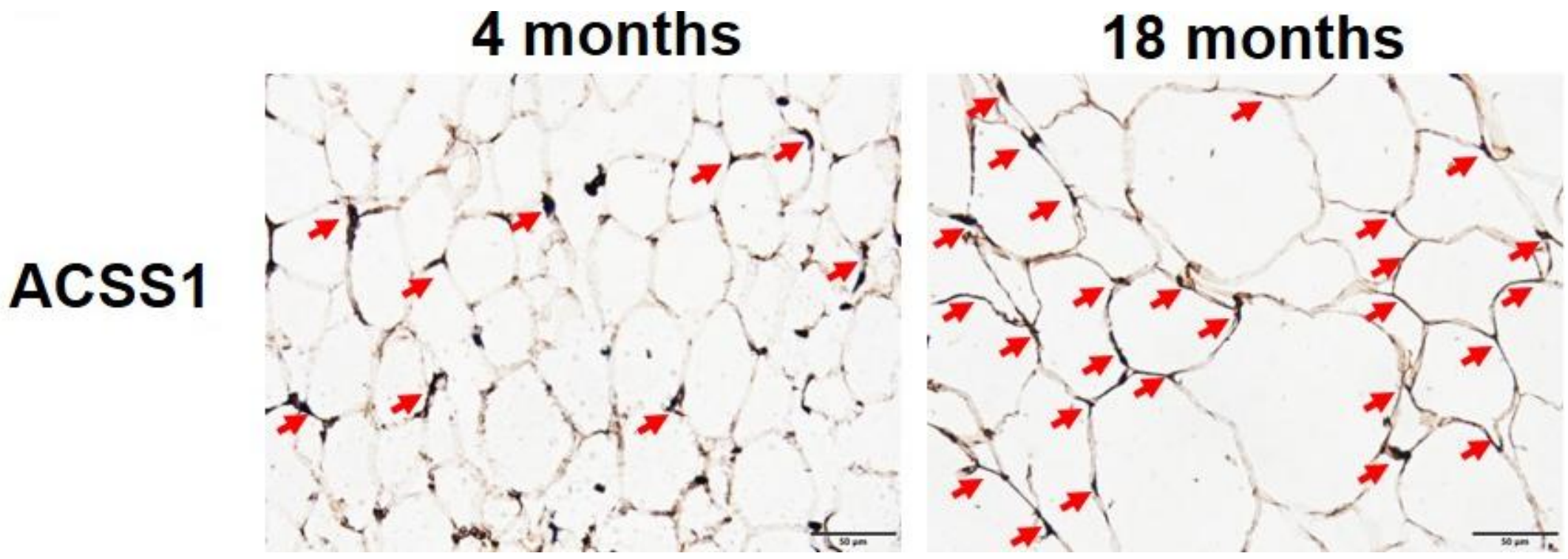

Fig. S2. Immunohistochemistry detection of ACSS1 in the mouse models.

Supplementary Figure S3. uncropped western blots

Figure 1E

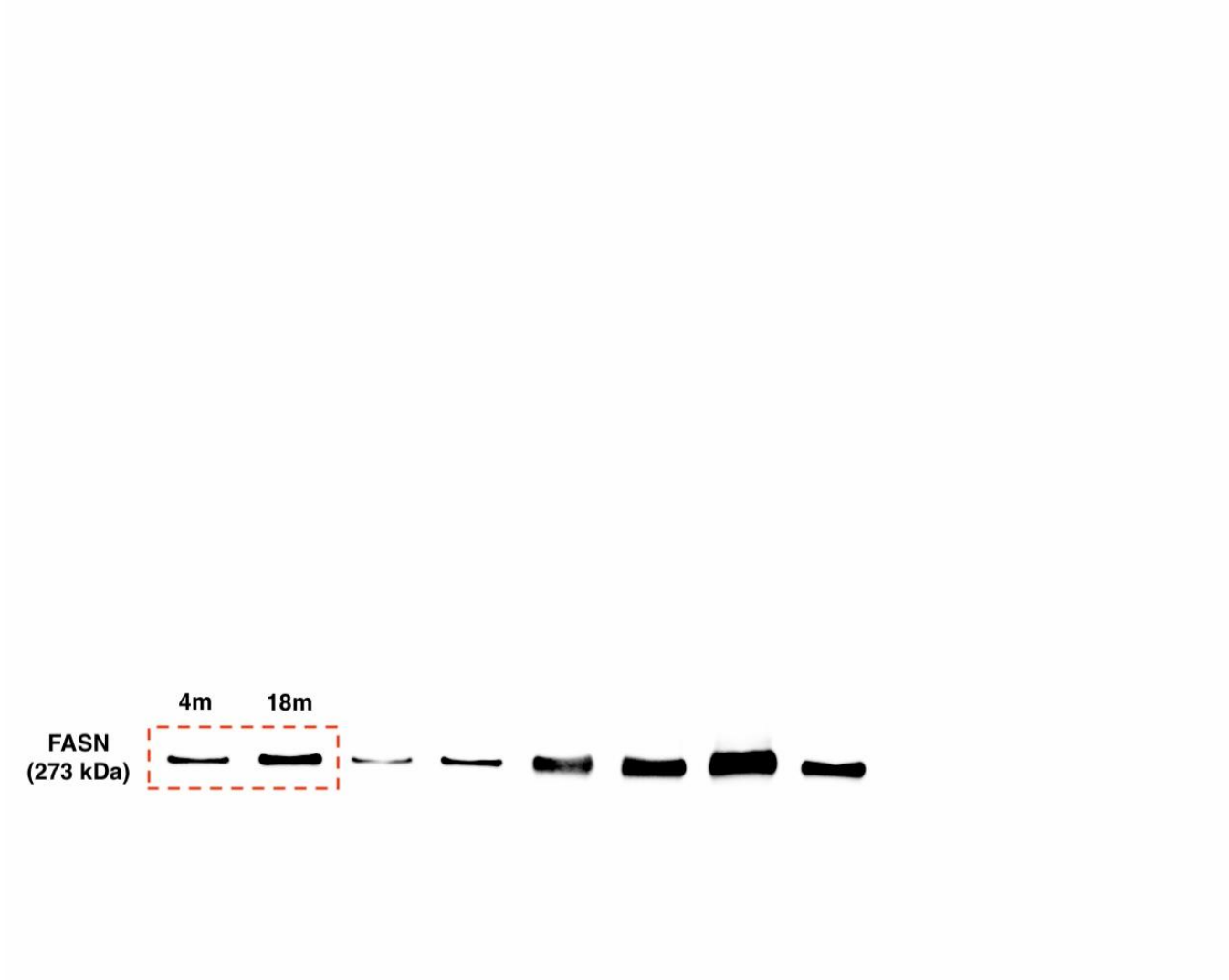

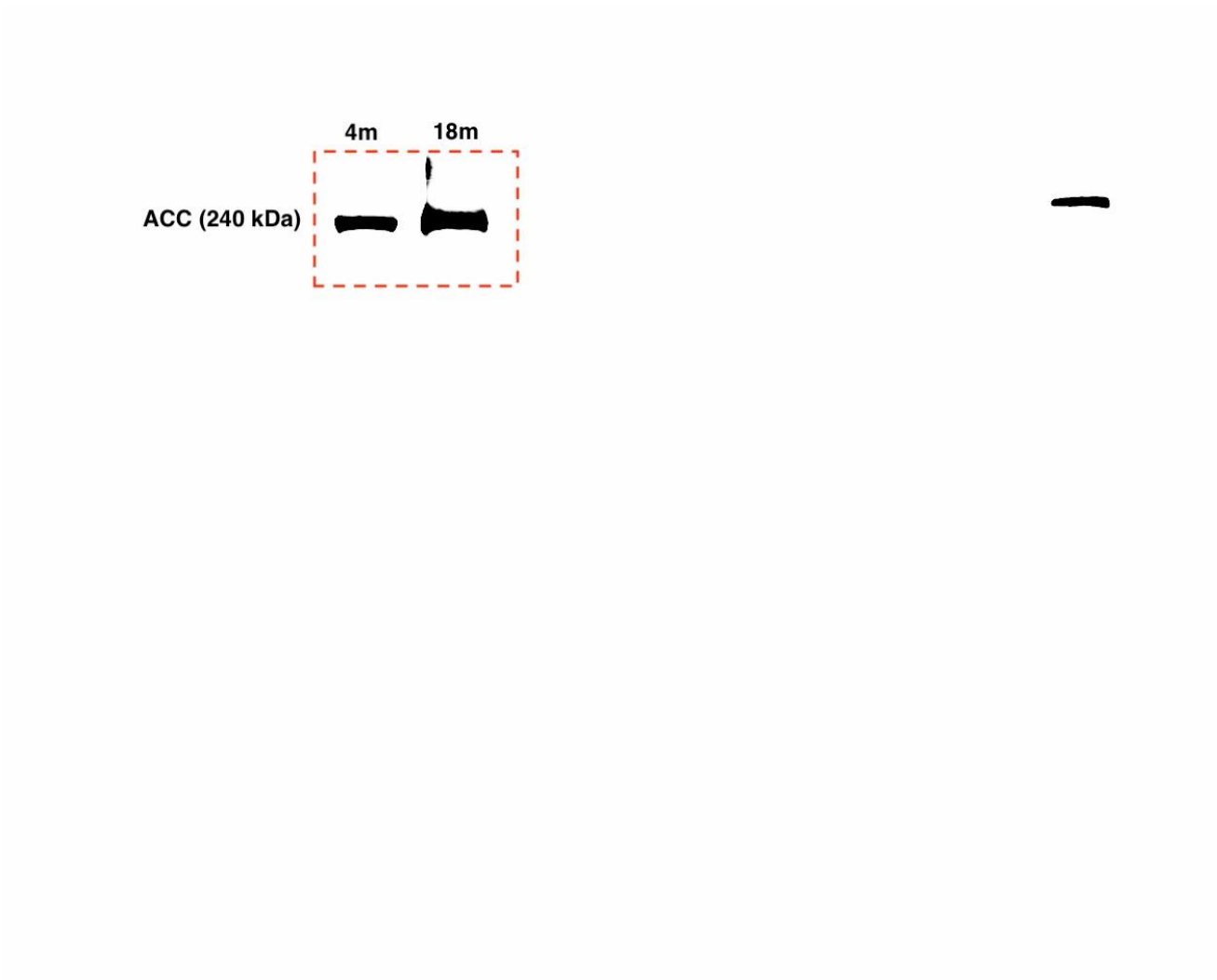

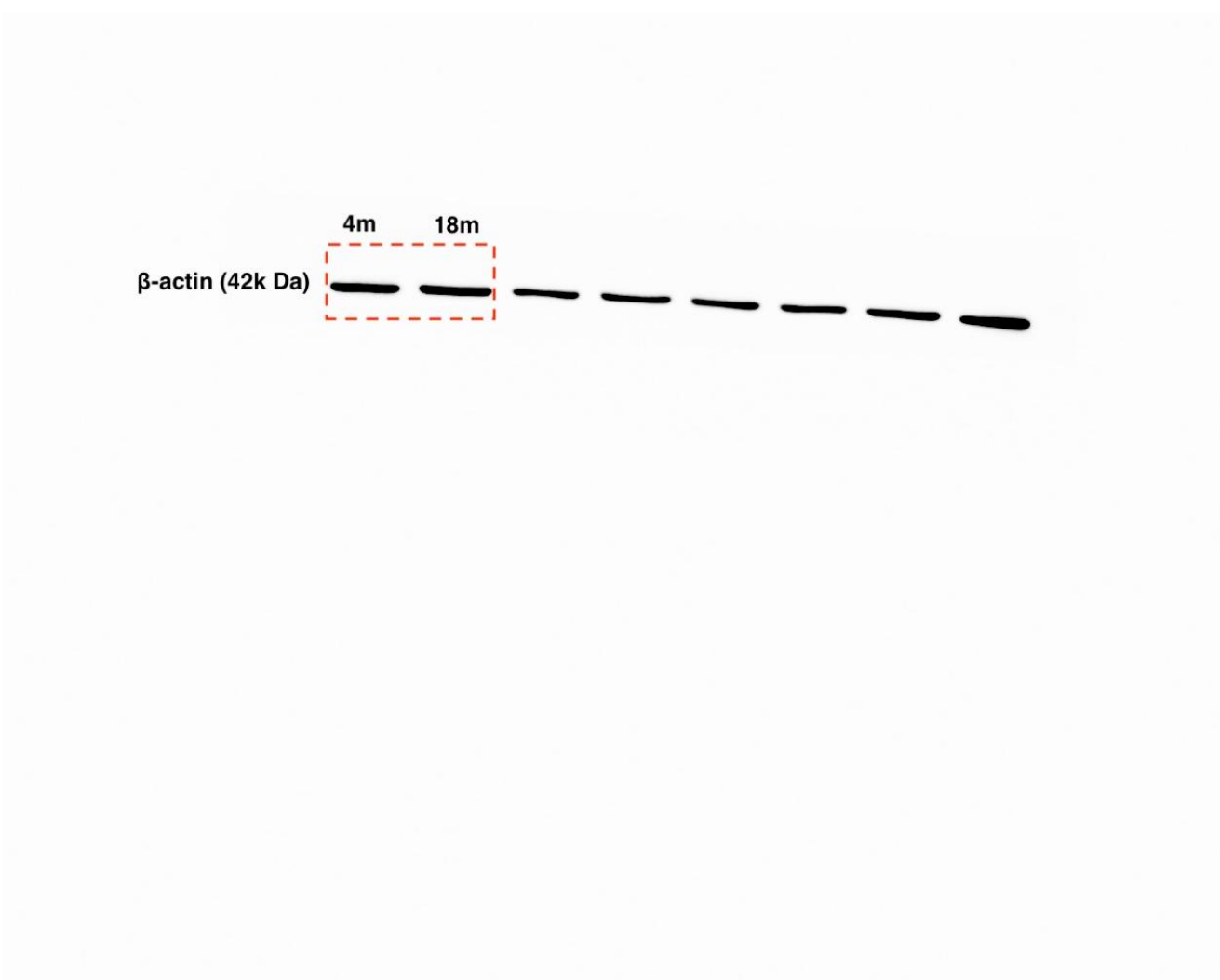

Figure 1H

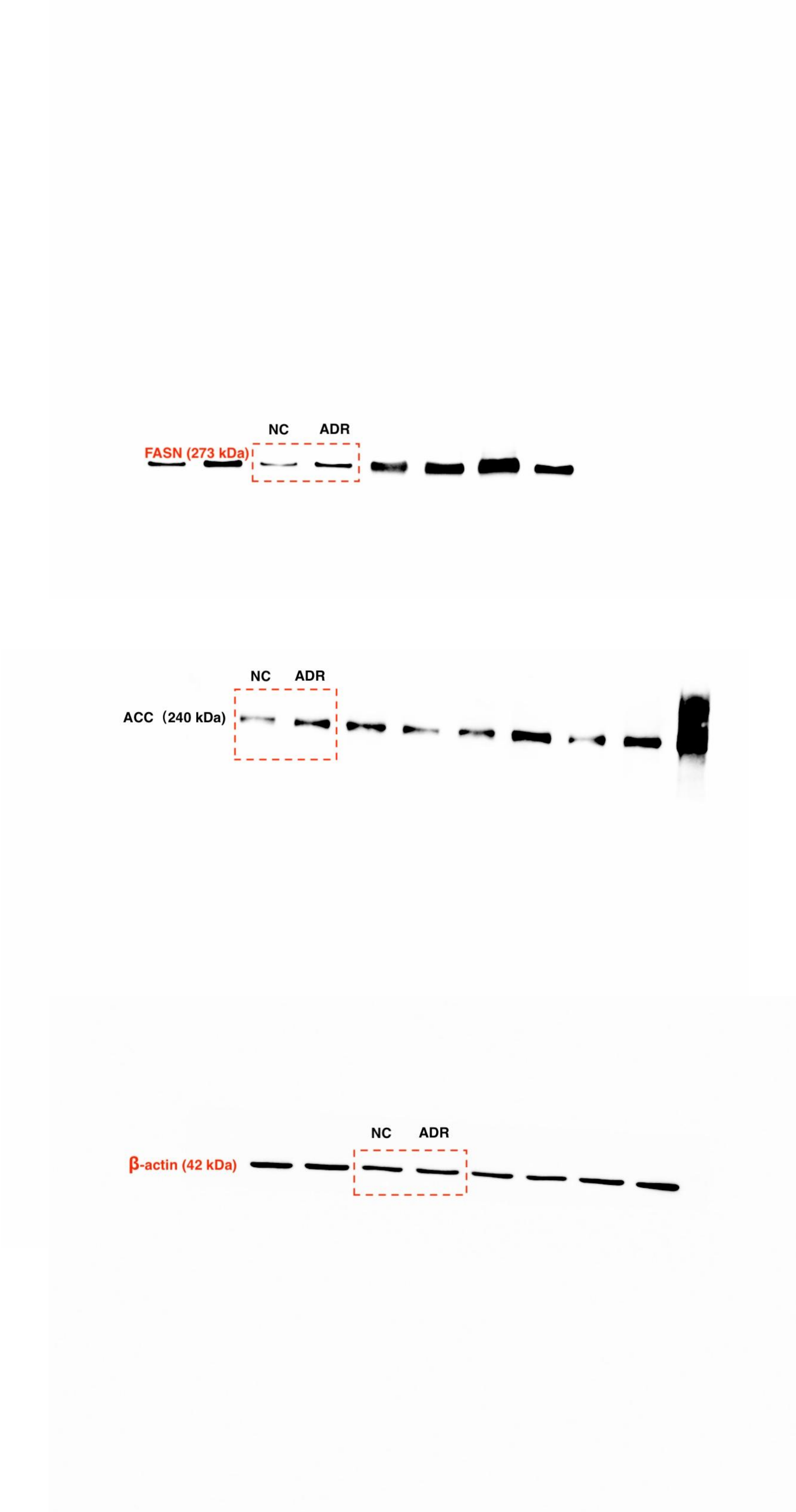

Figure 2H

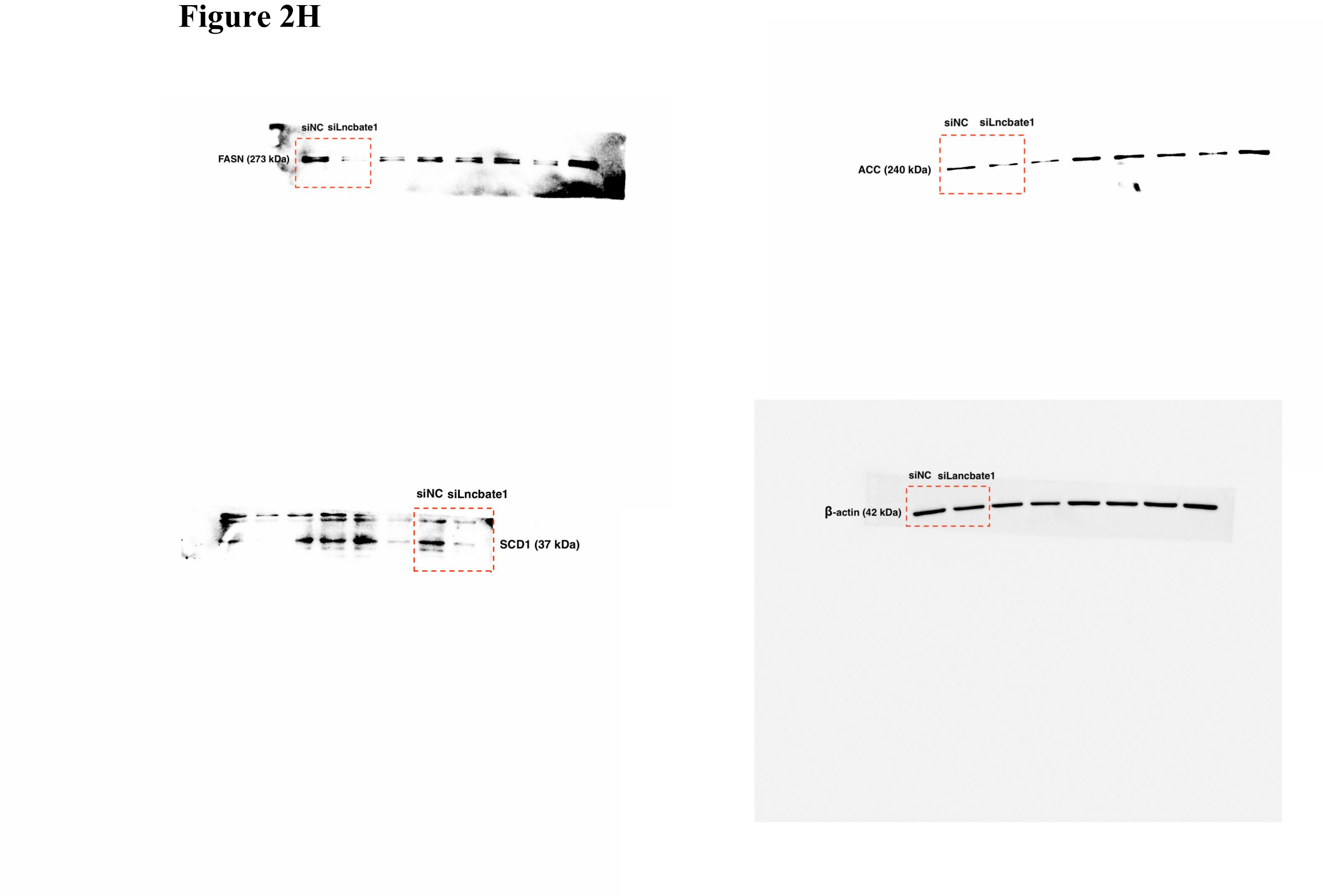
siNC siLncbate1
FASN (273 kDa)
siNC siLncbate1
ACC (240 kDa)
siNC siLncbate1
SCD1 (37 kDa)
siNC siLancbate1
β-actin (42 kDa)

Figure 4E

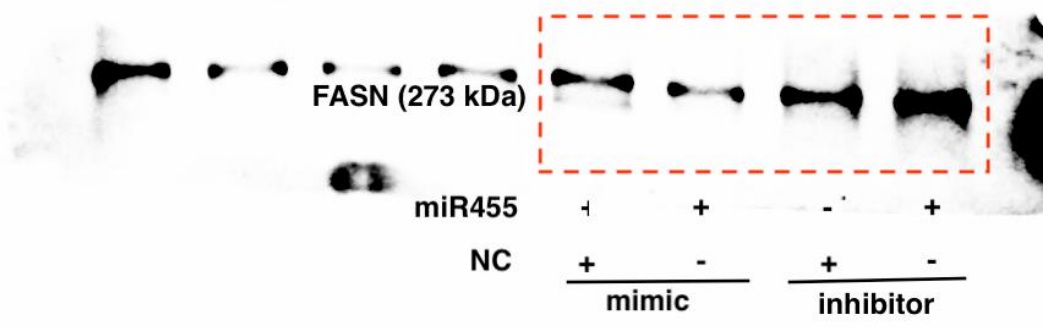
FASN (273 kDa)
miR455
NC
mimic
inhibitor

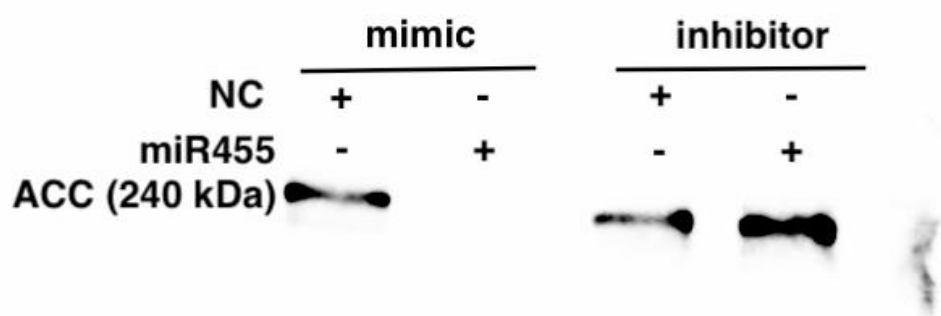
mimic
inhibitor
NC
miR455
ACC (240 kDa)

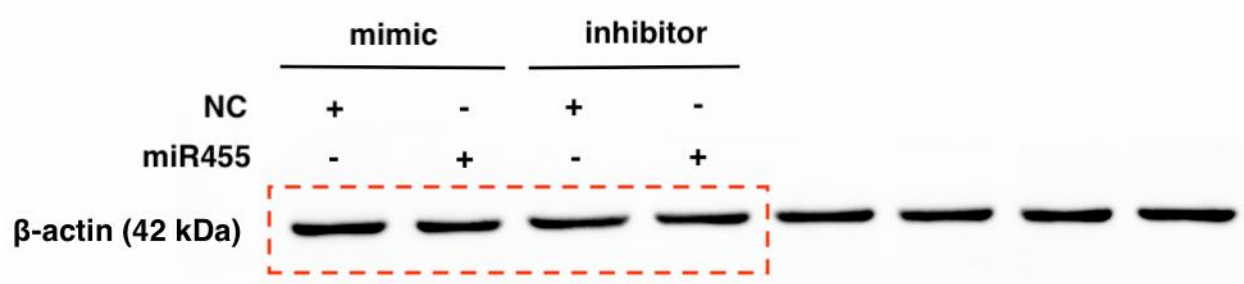
mimic
inhibitor
NC
miR455
β-actin (42 kDa)

Figure 5H

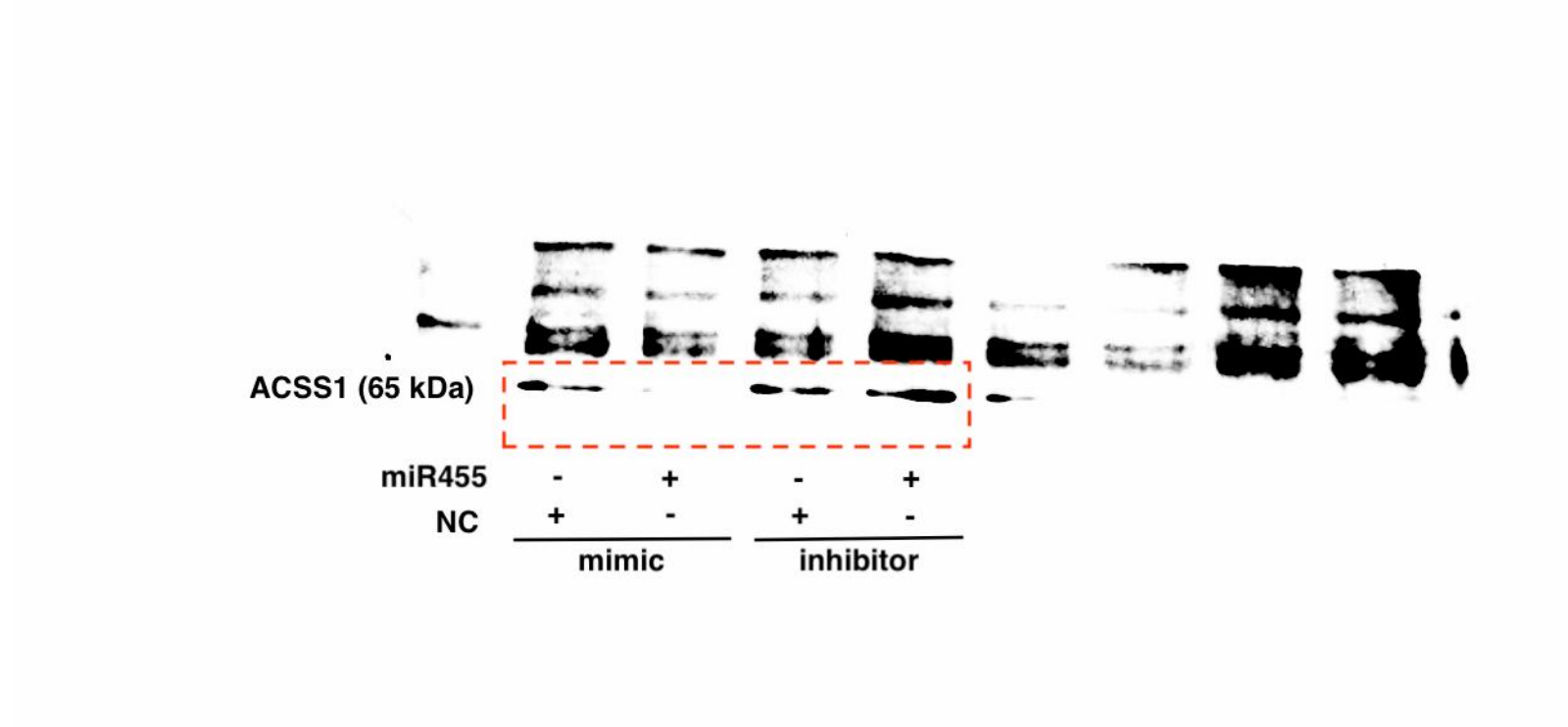
ACSS1 (65 kDa)
miR455
NC
mimic
inhibitor

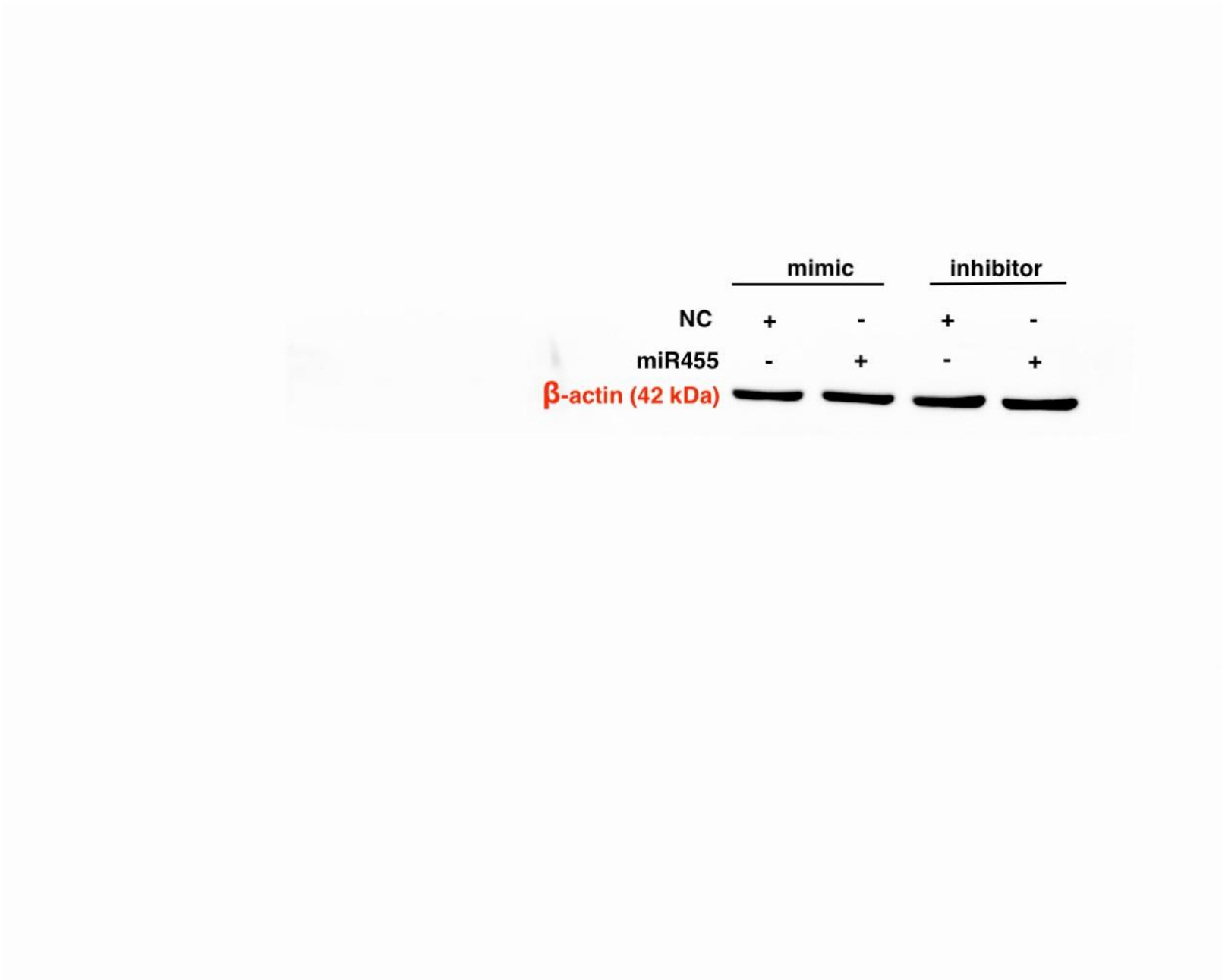
mimic
inhibitor
NC
miR455
β-actin (42 kDa)

Figure 6B

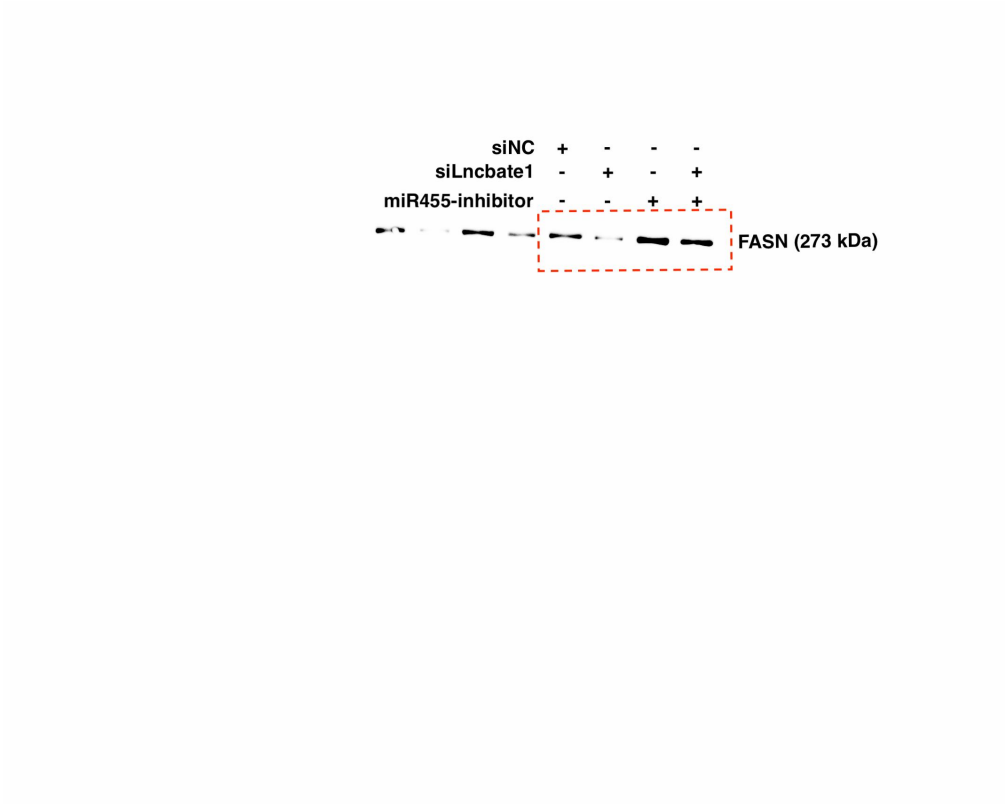
siNC + - - -
siLncbate1 - + - +
miR455-inhibitor - - + +
FASN (273 kDa)

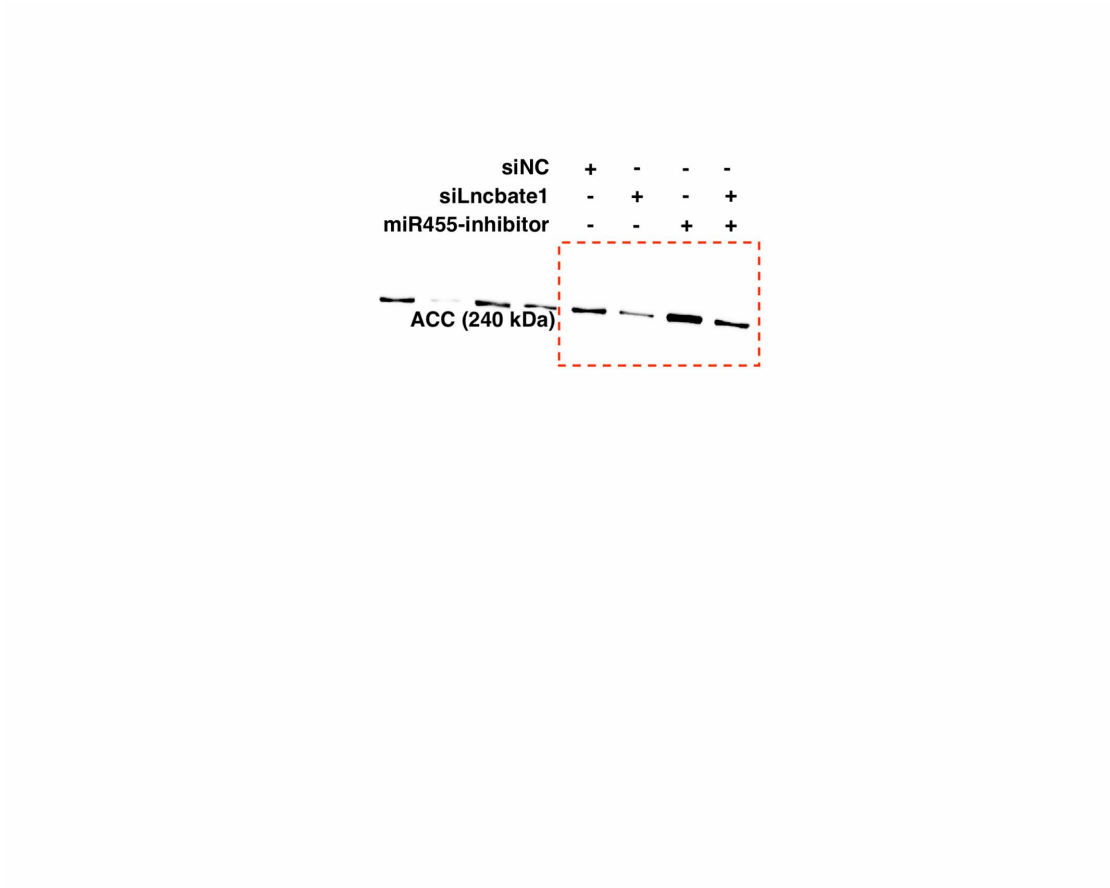
siNC + - - -
siLncbate1 - + - +
miR455-inhibitor - - + +
ACC (240 kDa)

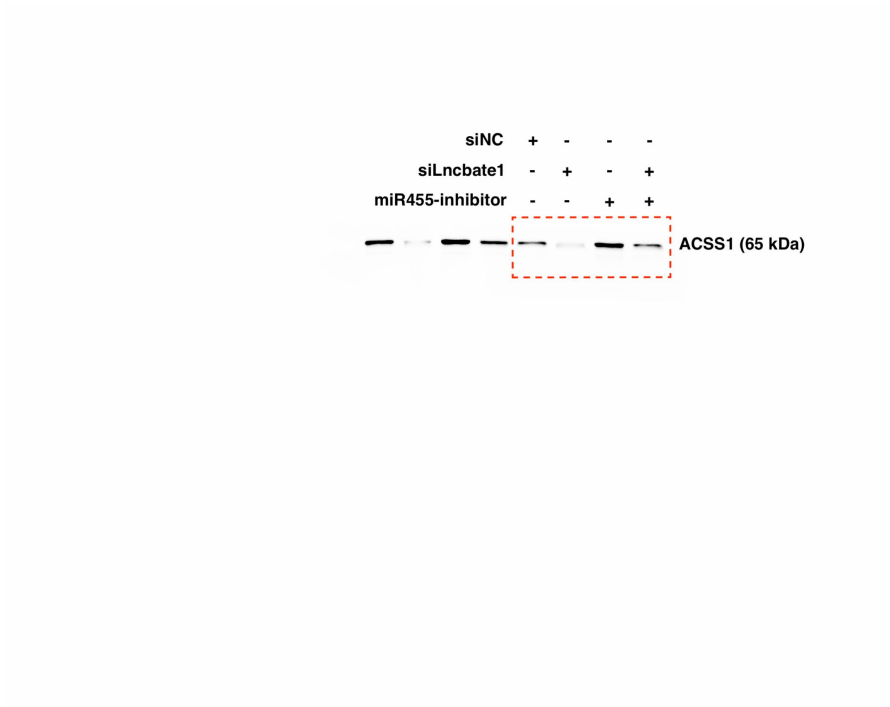
siNC + - - -
siLncbate1 - + - +
miR455-inhibitor - - + +
ACSS1 (65 kDa)

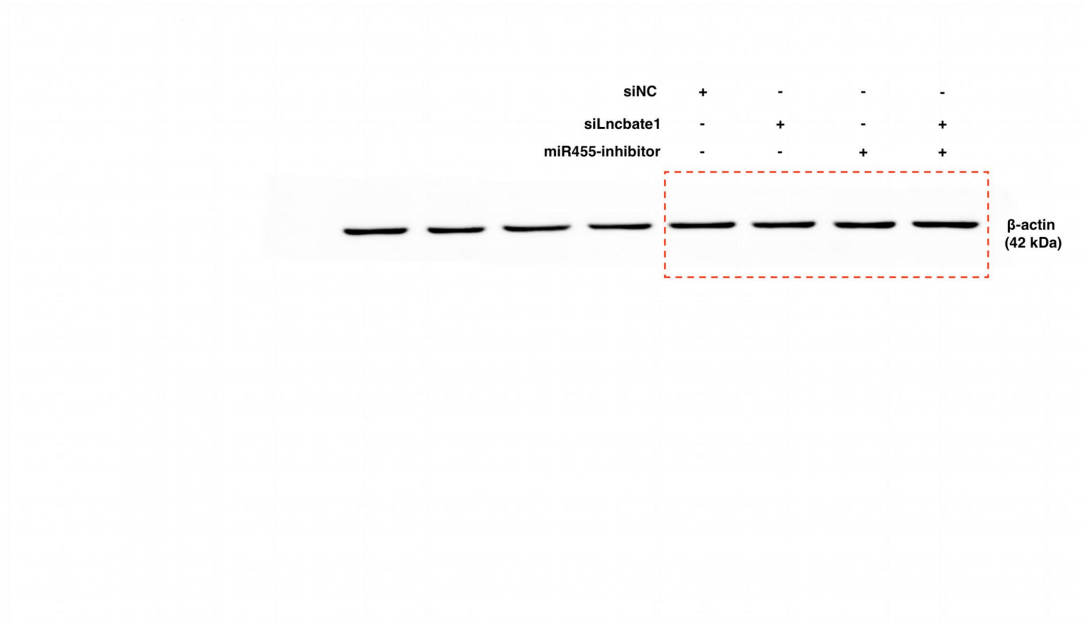
siNC + - - -
siLncbate1 - + - +
miR455-inhibitor - - + +
β-actin
(42 kDa)
